# Hepatitis B screening and knowledge among Chinese and Vietnamese students in Australia

**DOI:** 10.1371/journal.pone.0299224

**Published:** 2024-03-04

**Authors:** Elena Cama, Loren Brener, Timothy Broady, Robyn Horwitz, Defeng Jin, Hoang Minh Khoi Vu, K. O. E. Wu, Carla Treloar

**Affiliations:** Centre for Social Research in Health, UNSW Sydney, Sydney, NSW, Australia; Chinese Academy of Medical Sciences and Peking Union Medical College, CHINA

## Abstract

Research has shown that there are significant gaps in hepatitis B knowledge among migrant communities who are at risk of hepatitis B, such as Chinese and Vietnamese communities. Many students studying within Australia come from countries with high prevalence of hepatitis B. However, there is very little research examining hepatitis B knowledge, screening, or vaccination among university students in Australia or worldwide. The aim of this paper was to measure both levels of and demographic differences in hepatitis B screening and knowledge among Chinese and Vietnamese students in Australia. Online surveys were completed by 112 Chinese- and 95 Vietnamese-identifying students in Australia, measuring knowledge of hepatitis B, engagement in screening and vaccination, and demographic characteristics. Results show that although engagement in screening and vaccination for hepatitis B was high, there were significant gaps in knowledge around transmission of hepatitis B. There were also some key demographic differences in screening and knowledge. For instance, those born in Australia were more likely to have been screened compared to those born Mainland China, Hong Kong, or Vietnam. Chinese students born in Australia had lower levels of knowledge compared to those born in Mainland China or Hong Kong. Among both samples, knowing someone living with hepatitis B was associated with higher levels of knowledge. Findings underscore the need for education-based interventions to address the significant gaps that exist in knowledge around hepatitis B, with a specific need for culturally appropriate resources in a range of languages to cater to the diverse communities who may be at risk of hepatitis B.

## Introduction

Hepatitis B virus is an infection affecting the liver, which can be transmitted from person to person through bodily fluids such as blood. Infection is considered chronic when it has persisted for greater than six months, with chronic infection associated with hepatocellular carcinoma and higher mortality rates [1]. It is estimated that approximately 316 million people are living with chronic hepatitis B worldwide, and that hepatitis B-related diseases resulted in the deaths of 820,000 people in 2019. [2]. Hepatitis B is prevalent in East and South-East Asian countries, with estimates suggesting that death rates linked to hepatitis B are among the highest in the Western Pacific and South-East Asia regions [2]. An estimated 222,559 people are living with the virus in Australia [3], though only 73% are estimated to have been diagnosed [3, 4]. Research indicates that the majority of people living with chronic hepatitis B a were born overseas, with the most common regions being Northeast and Southeast Asia [3, 4]. People who are migrating to Australia from a country where hepatitis B is endemic are recommended to be tested for hepatitis B and assessed for vaccination status, with the recommendation being that catch-up vaccination for hepatitis B would be offered where required [5].

Broadly, there is some research to suggest that knowledge of hepatitis B is less than optimal among migrant communities in Australia [6–10]. In particular, there are knowledge gaps in relation to transmission of the virus, with misconceptions about the transmission of hepatitis B through food and water prevalent among migrant communities [6, 11–16]. Many students studying within Australia come from countries with high prevalence of hepatitis B. However, there is very little research examining hepatitis B knowledge, screening, or vaccination among university students in Australia or worldwide. The little research on hepatitis B knowledge among university students suggests that knowledge of the virus appears to be mixed. For instance, Ahmad and colleagues [17] examined hepatitis B knowledge among international students studying at a university in Malaysia and found that knowledge was low but was higher among participants who reported having a family member or relative living with the virus. Researchers in Lahore in Pakistan surveyed 2,800 students across six different universities and found that students had fair levels of knowledge, but that significant gaps existed particularly in relation to awareness of the hepatitis B vaccine [18]. Research among freshman in the Jiangsu province in China found that knowledge of the virus was low, particularly among students whose fathers had lower education levels [19]. In a study among university students in Scotland, including students from countries with high hepatitis B prevalence, students reported having low levels of knowledge and felt that their risk of acquiring the infection was low, but felt confident to access health care if needed [20].

Although there is limited research among students on hepatitis B generally, there is some research specifically examining levels of knowledge among health and medical students, given that this is a group who will finish university and enter into the health workforce treating people living with the virus [21–28]. However, it is possible that health and medical students may have greater knowledge of hepatitis B compared to the broader population of university students [e.g., see 18, 29–31]. A knowledge gap remains, therefore, on hepatitis B screening and knowledge among students from culturally and linguistically diverse backgrounds more broadly. The aim of this paper is to examine levels of hepatitis B screening and knowledge among Chinese and Vietnamese students in Australia, and to explore whether screening and knowledge varies according to demographic characteristics.

## Methods

### Sample and procedure

The data presented in this paper forms part of a larger research project that aims to monitor experiences and the expression of stigma towards people affected by blood borne viruses and sexually transmissible infections [32]. The data are from one component of the project, which surveyed students of Chinese and Vietnamese background about hepatitis B. Researchers of Chinese and Vietnamese background distributed advertisements for the survey on social media pages targeted at the Chinese and Vietnamese community. Prospective participants were directed to a survey website where they could find further information and complete the survey in English, simplified Chinese, or Vietnamese. Eligibility criteria for the study were that participants were over 18 years of age, Chinese or Vietnamese background, and currently studying in Australia at universities, TAFE, or English-language colleges. Participants consented to the study by ticking a checkbox at the beginning of the survey and by submitting their responses to the survey. The surveys took approximately 15–20 minutes to complete. Upon completion, each participant received an AUD $15 gift voucher as reimbursement for participation. Data collection took place between 16–30 November 2021. This research holds ethics approval from the UNSW Human Research Ethics Committee.

### Measures

#### Knowledge of hepatitis B

Three domains of hepatitis B knowledge were measured using 30 items adapted from previous research [6]. The domains were general knowledge of the virus (including awareness of vaccination and treatment), transmission of the virus, and prevention. General knowledge included items, ‘hepatitis B can only be identified by a blood test’ (True) and ‘there is a vaccination that can prevent hepatitis B infection’ (True). Transmission included items such as, ‘hepatitis B is caused by the hepatitis B virus’ (True) and ‘hepatitis B is caused by stress and negative emotions’ (False). Prevention included items such as ‘Someone can prevent themselves from getting hepatitis B, or giving it to others by avoiding blood-to-blood contact’ (True) and ‘Someone can prevent themselves from getting hepatitis B, or giving it to others by maintaining good hygiene (e.g., washing hands frequently, general cleanliness)’ (False). Available response options were ‘true’, ‘false’, and ‘unsure’, which were recoded into ‘incorrect’ (0) and ‘correct’ (1), with ‘unsure’ responses coded as ‘incorrect’. The items were summed to form an overall knowledge of hepatitis B scale, where higher scores indicated greater knowledge of the virus.

#### Hepatitis B screening and vaccination

Screening for hepatitis B was assessed using two items, which asked firstly if participants had ever been tested for hepatitis B and secondly whether they were tested in Australia. Participants were also asked if they had ever received the hepatitis B vaccine, and whether they had received the full course of vaccination.

#### Demographic characteristics

Questions measuring demographic characteristics were included in the survey, including age, gender, education, state of residence, and whether participants personally know someone living with hepatitis B.

*Data analyses*. Data were analysed using IBM SPSS Statistics software version 28. Descriptive statistics were used to describe the data, including demographic, hepatitis B screening and vaccination, and knowledge characteristics of the sample. Chi square tests were used to examine the association between screening and gender and country of birth, with Cramer’s V used to measure the strength of association. The same tests could not be conducted for differences in vaccination due to small cell sizes. Non-parametric Mann Whitney U tests were used to measure differences in knowledge for men and women, between those who knew someone and those who did not know someone living with hepatitis B, and between those born in Australia or overseas (in Mainland China or Hong Kong among the Chinese sample, and in Vietnam among the Vietnamese sample). Due to small numbers reporting non-binary and other terms for gender, comparisons could only be conducted between men and women.

## Results

There were 112 Chinese students and 95 Vietnamese students in the sample. The mean age among Chinese students was 24 years, while the average age among Vietnamese students was 23 years. In the Chinese student sample, half (51.8%) identified as male, while in the Vietnamese student sample, half (51.6%) identified as female. In both samples, around half of participants reported that they were born in Australia (51.8% of the Chinese sample vs 45.3% of the Vietnamese sample). While most students were studying at university, some were studying at a vocational education and training (VET) or technical and further education (TAFE) institute or at an institute for English language intensive courses. Additional demographic characteristics are presented in Table 1.

**Table 1:** Demographic characteristics of the student samples.

N (%)	Chinese students	Vietnamese students
	N = 112	N = 95
**Age Mean (SD), Range**	24.31 (3.16), 18–32	23.38 (2.69), 18–31
**Gender identity**		
Man/male	58 (51.8)	41 (43.2)
Woman/female	54 (48.2)	49 (51.6)
Non-binary	0 (0)	2 (2.1)
Different term	0 (0)	1 (1.1)
Prefer not to answer	0 (0)	2 (2.1)
**Enrolled in**		
University	104 (92.9)	79 (83.2)
VET/TAFE	5 (4.5)	13 (13.7)
ELICOS Institute	1 (0.9)	1 (1.1)
Other	2 (1.8)	2 (2.1)
**Country of birth**		
Australia	58 (51.8)	43 (45.3)
Mainland China	51 (45.5)	-
Hong Kong SAR	3 (2.7)	-
Taiwan	0 (0)	-
Vietnam	-	52 (54.7)
**Length of time in Australia**		
0–2 years	6 (5.4)	14 (14.7)
3–5 years	27 (24.1)	29 (30.5)
6–10 years	16 (14.3)	9 (9.5)
More than 10 years	63 (56.3)	43 (45.3)
**State of residence**		
Australian Capital Territory	17 (15.2)	9 (9.5)
New South Wales	35 (31.3)	23 (24.2)
Northern Territory	11 (9.8)	6 (6.3)
Queensland	13 (11.6)	13 (13.7)
South Australia	4 (3.6)	2 (2.1)
Tasmania	5 (4.5)	9 (9.5)
Victoria	17 (15.2)	21 (22.1)
Western Australia	10 (8.9)	12 (12.6)

Note: ELICOS refers to English Language Intensive Courses for Overseas Students.

Details about screening, vaccination, and information for hepatitis B among the student samples are presented in Table 2. Among both samples of students, self-reported testing for hepatitis B was high (above 70%), and most of those who had ever been tested reported that they had done so within Australia. Further, participants reported very high satisfaction rates (around 90%) with the information that they had received from health professionals at the time that they were tested. Vaccination against hepatitis B was also high, with 88.7% of Chinese students and 82.1% of the Vietnamese students reporting ever receiving the hepatitis B vaccine, and most of these students reporting receiving a full course of vaccination. Nearly one fifth of each sample reported that they know someone living with hepatitis B.

**Table 2:** Hepatitis B screening, vaccination, and information among the student samples.

N (%)	Chinese students	Vietnamese students
	N = 112	N = 95
**Ever tested for hepatitis B**		
No	13 (12.3)	20 (21.1)
Yes	82 (77.4)	68 (71.6)
Not sure	11 (10.4)	7 (7.4)
**Tested in Australia (among those tested)**	73 (89.0)	59 (86.8)
**Satisfied or very satisfied with information about hepatitis B received at time of testing (among those tested)**	76 (92.7)	61 (89.7)
**Ever had the hepatitis B vaccine**		
No	5 (4.7)	6 (6.3)
Yes	94 (88.7)	78 (82.1)
Not sure	7 (6.6)	11 (11.6)
**Received full course of vaccination (among those vaccinated)**	90 (95.7)	70 (89.7)
**Know someone living with hepatitis B**	19 (18.1)	17 (17.9)

The mean knowledge scores were 11.78 (SD = 3.86, Range 0–26) among the Chinese sample and 12.85 among the Vietnamese sample (SD = 4.67, Range 0–24), indicating that there were significant gaps in students’ knowledge of the virus. Students largely were aware that there was a vaccination available to prevent hepatitis B (88.8% Chinese students; 83.2% Vietnamese students). However, low proportions of students were aware that there are effective pharmaceutical medicines available to treat hepatitis B (28.0% Chinese students; 15.8% Vietnamese students). Participants held misconceptions about transmission of the virus, for example, many students incorrectly believed that hepatitis B could be spread through poor sanitation and hygiene (92.5% Chinese students; 87.4% Vietnamese students) and drinking too much alcohol (92.5% Chinese students; 75.8% Vietnamese students). Students also held misconceptions about prevention of hepatitis B, with large proportions of students incorrectly reporting that hepatitis B could be prevented through making sure food or water are not contaminated with hepatitis B (87.9% Chinese students; 89.5% Vietnamese students), maintaining good hygiene (e.g., washing hands frequently, general cleanliness) (92.5% Chinese students; 88.4% Vietnamese students), avoiding sharing eating utensils with a person who has hepatitis B (89.7% Chinese students; 87.4% Vietnamese students), and exercising (89.7% Chinese students; 88.4% Vietnamese students). The proportions of students who incorrectly responded to items that reflected how hepatitis B could be transmitted were much lower. For instance, only some participants incorrectly believed that hepatitis B could **not** be prevented through avoiding blood-to-blood contact (12.1% Chinese students; 12.6% Vietnamese students), using condoms when having sex (18.7% Chinese students; 28.4% Vietnamese students), not sharing any equipment for injecting drugs (15.0% Chinese students; 13.7% Vietnamese students), and having hepatitis B vaccinations (12.1% Chinese students; 14.7% Vietnamese students).

There were no significant differences in screening according to gender and knowing someone living with hepatitis B in either the Chinese or Vietnamese student samples. There was an association between screening and country of birth among the Chinese sample, χ^2^_(1)_ = 6.09, *p* = .014. The strength of the association was small to moderate, Cramer’s V = .253 [33]. Data showed that a higher proportion of participants born in Australia reported having ever been screened for hepatitis B compared to those who were born in Mainland China or Hong Kong (94.2% vs 76.7%). There was also an association between screening and country of birth among the Vietnamese sample, χ^2^_(1)_ = 11.88, *p* < .001. The association was moderately strong, Cramer’s V = .367 [33]. Data showed that a higher proportion of participants born in Australia reported having ever been screened for hepatitis B compared to those who were born in Vietnam (93.0% vs 62.2%).

There were no significant differences in knowledge by gender among the Chinese student sample. However, women had significantly higher knowledge of hepatitis B compared to men among the Vietnamese student sample (Median 13 vs 11) (*U* = 1,272.50, *z* = 2.18, *p* = 0.029). Among both samples, participants who knew someone living with hepatitis B had higher knowledge than those who did not (Chinese students: Median 13 vs 11, *U* = 1,007.50, *z* = 3.12, *p* = 0.002; Vietnamese students: Median 17 vs 11, *U* = 1,057.50, *z* = 3.97 *p* < 0.001). Among Chinese students, those who were born in Mainland China or Hong Kong had higher levels of knowledge than those born in Australia (Median 13 vs 11) (*U* = 1,803, *z* = 2.34, *p* = .019). There was no difference in knowledge according to country of birth among Vietnamese students.

## Discussion

The aim of this study was to examine levels of hepatitis B screening and knowledge among samples of Chinese and Vietnamese identifying students living in Australia, and to explore some of the demographic differences in levels of screening and hepatitis B knowledge. Findings from this study indicate that among these students, screening and vaccination rates for hepatitis B were quite high, with over 70% of both samples having ever been tested for the virus and over 80% reporting that they had ever received the vaccine. In both student samples, those who were born in Australia were more likely to report ever being screened for hepatitis B compared with those born in Mainland China, Hong Kong, or Vietnam. Although screening and vaccination (where required) for hepatitis B are recommended for overseas born people migrating to Australia, this may not always occur. Thus, this highlights the importance of advocating for the importance of screening among people migrating to Australia.

Although many of the students in this study reported that they had been screened (above 70%) and vaccinated (above 80%), the data from this study show that there were significant gaps in hepatitis B knowledge among these students, particularly in knowledge around transmission of the virus. These findings are consistent with previous research which has found that significant gaps in knowledge, particularly around transmission, of hepatitis B exist in migrant communities generally, suggesting that these gaps persist among students [6, 11–16]. Within the knowledge scale, there were 13 items which assessed knowledge around prevention of hepatitis B. These items measured whether students believed that hepatitis B could be prevented using a range of strategies, including avoiding blood-to-blood contact, using condoms when having sex, not sharing equipment for injecting drugs, not drinking alcohol, exercising, avoiding eating food prepared by a person infected with hepatitis B, avoiding sharing eating utensils with a person who has hepatitis B, having hepatitis B vaccinations, taking traditional medicine, and maintaining good hygiene. Five of the thirteen items included were true, and findings indicated that the majority of Chinese and Vietnamese students understood that hepatitis B could be prevented through strategies such as avoiding blood-to-blood contact, using condoms when having sex, and having hepatitis B vaccinations.

However, significant proportions of students incorrectly believed that hepatitis B could also be prevented by exercising, making sure food or water are not contaminated with hepatitis B, and maintaining good hygiene (e.g., washing hands frequently, general cleanliness). Misperceptions around transmission of hepatitis B particularly through contaminated food and water may reflect conflation of the different types of viral hepatitis, as has been found among other studies among migrant groups [34]. One quarter of Chinese students (n = 28, 25%) and nearly one fifth of Vietnamese students (n = 17, 17.9%) reported that they believed all thirteen items to be true. This could reflect straightlining of responses (providing the same answers in matrix tables to get through responses quickly) or that participants were guessing that all responses were true. Alternatively, this could be indicative of students believing that taking a holistic approach to health can help to prevent illness. This is in keeping with holistic approaches to health and illness reflected in the cultural beliefs of Chinese and Vietnamese communities and in complementary and alternative medicine, which focus on concepts of balance and harmony, which can be achieved through factors such as exercise, nutrition, and internal peace [35, 36]. For instance, researchers investigating hepatitis B knowledge among Chinese and Vietnamese people living in Australia have suggested that misperceptions in knowledge might instead reflect cultural beliefs whereby emotional and mental wellbeing are considered key to good health [6, 36].

There were also some notable demographic differences in levels of knowledge among these students; namely that Chinese and Vietnamese students who knew someone living with hepatitis B had higher knowledge than those students who did not know someone living with hepatitis B. This is consistent with a small amount of research on knowledge among general Chinese and Vietnamese communities [6, 7, 17]. Chinese students who were born in Mainland China or Hong Kong also had higher levels of knowledge compared to those born in Australia, but these geographic differences did not exist among the Vietnamese student sample. It is possible that Chinese students born in Mainland China or Hong Kong had greater knowledge due to the higher prevalence of hepatitis B.

It is evident that education-based interventions are required to improve knowledge of hepatitis B among migrant communities, including students. It is especially important that such interventions are culturally appropriate, sensitive, and in a range of languages. In a study among the Vietnamese community, Vu and colleagues [9] found that the majority of participants would prefer to receive information about hepatitis B in the Vietnamese language. Thus, it is essential that the development of such materials is done with consultation with community and in a variety of languages to cater to linguistically diverse populations. Further, there is evidence that education-based interventions can improve hepatitis B knowledge among migrant communities [11, 37–39]. Educational institutions may be one setting in which researchers can implement and evaluate interventions to improve knowledge of hepatitis B. Given that we know that misinformation and lack of knowledge about the virus are associated with hepatitis B-related stigma [6, 40, 41], this highlights the importance of developing interventions that address both misinformation surrounding the virus as well as negative attitudes towards people living with the virus. For instance, there is evidence that brief online interventions involving some level of contact with people living with hepatitis B can improve attitudes towards people living with the virus among the general community [42]. It would be worthwhile to develop interventions in a range of languages for migrant communities and evaluate their effectiveness in addressing negative attitudes among these communities.

This research is limited in that it is a small, cross-sectional, and self-selecting sample of Chinese and Vietnamese students. This sample is not representative of Chinese and Vietnamese identifying students in Australia, and we note that we had no means of verifying the inclusion criteria for the study. The data is also limited in that it relies on self-reported data from participants on hepatitis B screening and vaccination, and it is possible that participants may not accurately recall screening and vaccination history or may conflate hepatitis A and B. We were unable to examine whether there was a difference in vaccination for hepatitis B according to country of birth, and we were also unable to examine whether there was a difference in levels of hepatitis B knowledge according to hepatitis B status, given that the numbers of students who reported that they are living with hepatitis B were very small. However, previous research indicates that there may not be any differences in knowledge among students based on hepatitis B status [43]. Still, this is an avenue for future research in the Australian context. We also did not collect data on the field of study among these student samples, and thus could not look at the differences in levels of knowledge among those studying in health or medical related fields. However, previous research has found that knowledge is likely to be lower among those who are studying in non-health or medical related fields [e.g., see 18, 29–31].

Findings from this study highlight that significant gaps in hepatitis B knowledge exist, particularly in relation to transmission of hepatitis B, among these samples of Chinese and Vietnamese students. This is concerning, given that there was still 20–30% of both samples who reported that they had never been tested for hepatitis B or were not sure if they had been tested, and more than 10% of both samples had never had the vaccine or were not sure. Students may be a population who can give insight about levels of knowledge within their broader communities and highlight where efforts should be made to implement informational and stigma-based interventions. Educational institutions may be a good setting within which to implement such interventions to try to improve hepatitis B knowledge, which may have flow on effects in increasing access to testing, vaccination, and linkage to care, as well as reducing any negative attitudes towards those living with hepatitis B.
